# Systematic Review of Potential Occupational Respiratory Hazards Exposure Among Sewage Workers

**DOI:** 10.3389/fpubh.2021.646790

**Published:** 2021-03-08

**Authors:** Kamarulzaman Muzaini, Siti Munira Yasin, Zaliha Ismail, Ahmad Razali Ishak

**Affiliations:** ^1^Department of Public Health Medicine, Faculty of Medicine, Universiti Teknologi MARA, Sungai Buloh, Malaysia; ^2^Centre of Environmental Health and Safety, Faculty of Health Sciences, Universiti Teknologi MARA, Puncak Alam, Malaysia

**Keywords:** respiratory hazards, sewage workers, sewage plants, respiratory symptoms, pulmonary function

## Abstract

**Background:** Sewage workers have a higher risk of exposure to various potential occupational respiratory hazards found in sewage plants. Although previous studies discuss occupational respiratory hazard concentration impacting sewage workers' respiratory health, the results are scarce and mixed. Hence, there is a need to identify the potential respiratory hazards in sewage plants so as to clarify the short- and long-term respiratory health effects. Therefore, this systematic review (SR) aims to critically review previous studies investigating potential respiratory hazards found at sewage plants and their effects on sewage workers' respiratory health.

**Methods:** An SR was conducted using PubMed, EBSCO Medline, Web of Science, Scopus, and Google Scholar on peer-reviewed studies published between January 1994 and October 2020 evaluating the impact of potential exposure to respiratory hazards and its effects on respiratory health among sewage workers. “Sewage treatment plant,” “respiratory hazards,” and “respiratory health effects” were the three main search terms chosen in this SR. The inclusion criteria were (1) studies on potential occupational respiratory hazard exposure among sewage workers, (2) manuscripts written in English, and (3) studies published in the peer-reviewed literature. The human observational studies' quality was assessed using the Effective Public Health Practice Project Quality Assessment Tool.

**Results:** We identified 5,660 articles through an initial database search. Only 26 items met the inclusion criteria and were included in this review; 15 human observational studies and 11 environmental assessment studies were conducted in the sewage industries. Most of the human observational studies were rated as moderate quality, two studies were rated as weak quality, and one study with strong quality was identified. Hydrogen sulfide, bioaerosols, particulate matter 2.5 (PM 2.5), and volatile organic compounds (VOC) were found to be potential respiratory hazards. Most of the risks contributed to adverse outcomes on the sewage workers' respiratory health with some inconsistent findings on the relationship between respiratory hazard exposure and respiratory health effects.

**Conclusion:** Our review finds that, although this area is of great importance, quality studies are still lacking. There is a need for additional studies to clarify the effects of respiratory hazard exposure on sewage workers and respiratory health, especially PM 2.5 and VOC.

## Introduction

Various studies widely demonstrate that sewage treatment plants (STP) produce bundles of occupational hazards through different sewage plant processes to remove contaminants from wastewater or as by-products (1, 2). Occupational hazards can be described as “aspects of one's occupation-specific context that increase the risk of injury” (3). Occupational hazards refer to the potential risks to the health and safety of those people who work outside the home. Generally, there are several occupational hazards that potentially exist in the sewage plant, such as chemical, biological, and physical hazards (4). Exposure to these potential occupational hazards can lead to work-related diseases and adverse health effects. As a result, much previous literature shows that workers at sewage treatment plants are at high risk of experiencing a broad range of adverse health impacts, including respiratory disorders (asthma or chronic obstructive pulmonary disease), infections (such as tuberculosis, leptospirosis, hepatitis A, or tetanus), gastrointestinal problems (for example, gastroenteritis), skin illnesses (for instance, contact dermatitis or eczema), cancers (such as lung, stomach, and renal cancers), and general symptoms (such as unusual tiredness and headache) (4–6).

Occupational lung diseases are among the leading health impacts because sewage workers are likely to be exposed to various occupational respiratory hazards ranging from specific chemical agents to microbiological agents (7). Generally, occupational respiratory hazards can be present in several forms at any industry's workplaces, for instance, gases, dust, fumes, mists, vapors, smoke, fog, and sprays. Some substances are generated via industrial processes, for example, those tailored to sewage industries, such as during the aeration process, drying the sludge, and mechanical filtering processes. Sewage workers exposed to occupational respiratory hazards for a significant amount and duration can develop adverse respiratory health effects without proper hazard control strategies and personal protective equipment (8). These are usually due to infection, inflammation, and chemical sensitization along the airway tract and allergic responses (9).

To date, occupational lung diseases have become a global issue and have been researched extensively as they act as a significant contributor to morbidity and mortality. Based on the Global Burden of Disease Study (10), occupational respiratory hazard exposure is a crucial determinant of chronic, work-related respiratory disease. Also, it accounted for more than 500,000 mortality incidents and 13 million disability-adjusted life years in 2016 from chronic respiratory disease due to occupational respiratory hazard exposure (10). Besides that, the American thoracic society finds that the highest number of occupational diseases frequently recorded among sewage workers were respiratory (66%) followed by skin problems (31%) and noise-induced hearing impairment (11, 12).

Nowadays, the development of sewage plants has resulted in new technologies. Different processes of treating wastewater could contribute to further production and concentration of toxic air pollutants (13, 14). Even though several previous studies measured occupational respiratory hazard (i.e., hydrogen sulfide, endotoxins, inhalable dust) concentrations and the exposure effects on sewage workers' respiratory health, the results are scarce and mixed. The potential impacts of mixed gas exposure and dose-related effects could attenuate this issue further.

### Objectives

As such, the purpose of this systematic review is to identify potential occupational respiratory hazard exposure among sewage workers that could arise from sewage treatment plants and its effects on sewage workers' respiratory health with the intention that, in the future, this review may provide input to the authorities to plan several strategies to prevent and minimize as much as possible sewage workers' respiratory health effects from the identified occupational respiratory hazard exposures. This review extensively examines published studies of potential occupational respiratory hazard exposure among sewage workers and its impact on their respiratory health. Thus, this review includes articles from human observational studies examining the respiratory health effects of potential occupational respiratory hazard exposure. Environmental assessment studies are also included to measure the occupational respiratory hazard concentrations produced at sewage plants.

### Research Question

The key question of interest is the following: What are the potential occupational respiratory hazards that can be found at sewage plants and their effects on sewage workers' respiratory health?

## Methods

### Eligibility Criteria

A broad systematic review was conducted based on the Preferred Reporting Items for Systematic Reviews and Meta-Analyses (PRISMA) guidelines. We developed several eligibility criteria to address these critical question: What evidence of potential occupational respiratory hazards could arise from the sewage treatment plant and possibly cause adverse respiratory health effects among sewage workers? To widen our findings, we selected (i) observational studies involving human subjects and (ii) environmental assessment studies conducted in sewage plants. Both types of studies need to be undertaken in the sewage industry settings. The inclusion criteria were (i) studies on potential occupational respiratory hazard exposure among sewage workers, (ii) manuscripts written in English, and (iii) studies published in the peer-reviewed literature from January 1994 to October 2020. Meanwhile, the exclusion criteria were in the categories of (i) review articles and (ii) case report or forensic studies.

### Search Strategy

Comprehensive database searches were conducted between May and October 2020. All the relevant kinds of literature were extensively searched via five databases: PubMed, EBSCO Medline, Web of Science, Scopus, and Google Scholar. The search strategy was based on implementing the population, intervention, comparison, and outcomes (PICO) model developed first for MEDLINE and later adapted for other databases. The PICO approach utilized to guide us in constructing the organization and syntax of search terms was as follows: For population, we only selected sewage workers and studies done in sewage plants. For intervention, no interventional studies were expected in this study area given the observational nature of potential occupational respiratory hazard exposure among workers in sewage plants. For control/comparison, any comparison method could be included; it was not limited or specified provided the specific details of potential occupational respiratory hazard exposure monitoring and/or respiratory health effects among the study participants were reported. For outcomes, we looked into sewage workers' respiratory health effects from the exposure of potential occupational respiratory hazards that exist in sewage plants. Thus, the main search terms chosen were sewage treatment plant, respiratory hazards, and respiratory health effects. We used several search terms, such as “sewage treatment plant” well-defined with synonym “wastewater treatment plant.” Next, for the terms of respiratory health effects, we included several search phrases to cover the range of topics (namely, respiratory effects, respiratory symptoms, breathing, lung, pulmonary, and respiration). The literature search was expanded by including all combination pairs of the three main search terms.

Provided below is a search strategy sample from the PubMed online database:

(1) (Wastewater treatment plant OR sewage plant) AND (respiratory effects OR respiratory Symptoms OR breathing OR lung OR pulmonary OR respiration) AND hazards.

After completing the searches and excluding duplicate studies, two of the reviewers (KM and SMY) independently screened the identified articles' titles and abstracts to select relevant articles to be included for a full review. They also reviewed citations to seek several potential and relevant articles for inclusion. In the event that there was a difference of view or opinion for the study in fulfilling the inclusion or exclusion criteria, the third and fourth reviewers (ZI and ARI) engaged to resolve those issues.

**Figure 1:**
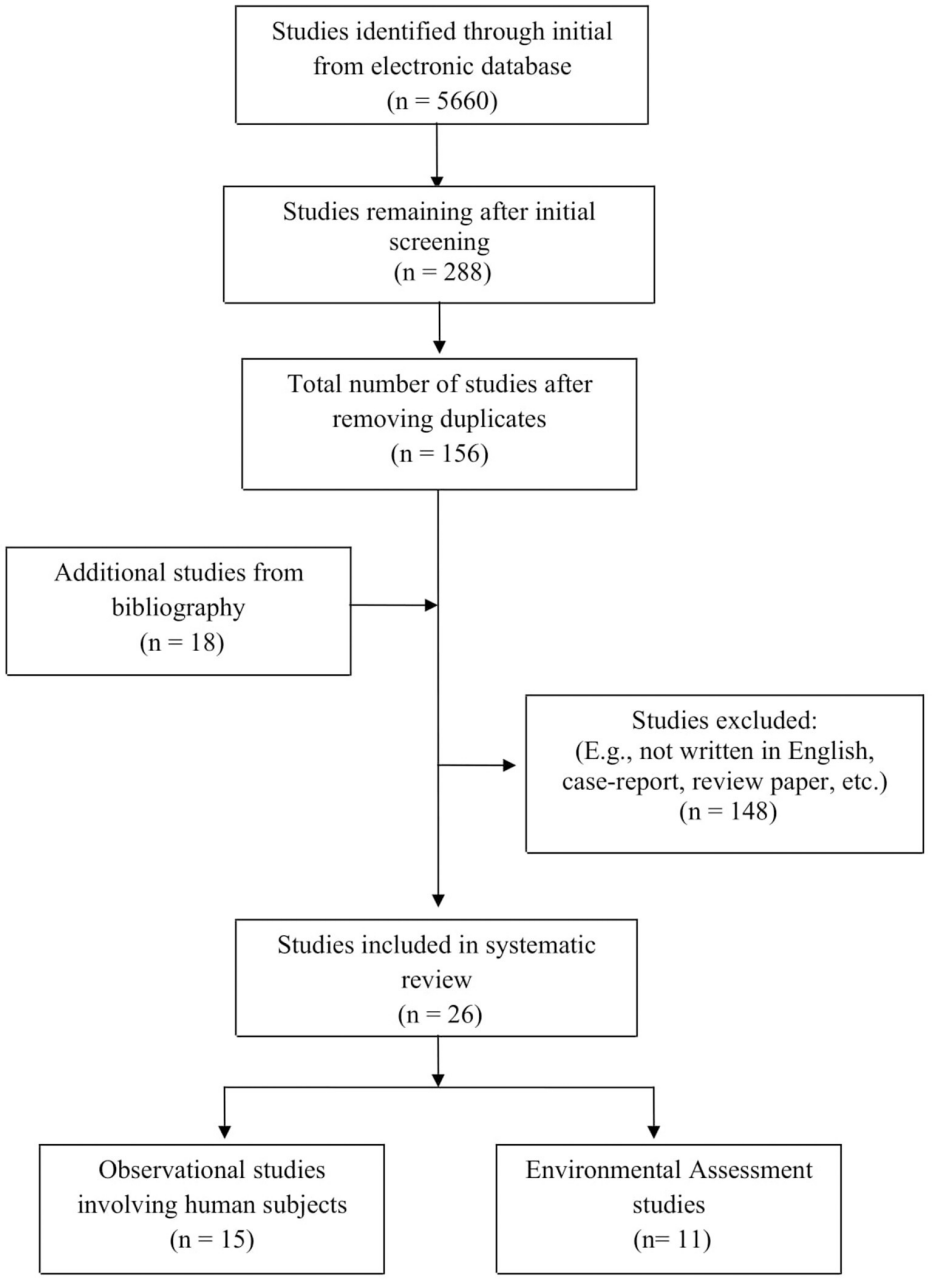
Literature search strategy and selection process for included studies (*n* = 26).

A difference in opinion occurred with regards to the suitability of selecting environmental assessment studies to be included in this review apart from human observational studies. Therefore, the reviewers decided to select only the environmental assessment studies that were conducted in sewage plants and assessed the related potential occupational respiratory hazards. Next, the articles chosen for full review were rescreened to ensure that the inclusion criteria were met. All the results were then transferred to the reference management software Endnote X7 to help the authors systematically manage the manuscripts and retrieve full-text articles. A flowchart of the literature search is shown in Figure 1.

### Quality Assessment

The human observational and environmental assessment studies' quality was assessed using the Effective Public Health Practice Project (EPHPP) quality assessment tool for quantitative studies (see **Tables 3**, **4**) (41), which evaluates the following items: selection bias, study design, confounders, data collection methods, and withdrawal/dropout rate. Guidance for article bias ratings was taken from the EPHPP quality assessment tool dictionary. The descriptions of the mentioned items are summarized as follows: Selection bias considers to what extent study participants are likely to be representative of the target population as well as the proportion of selected individuals who agree to participate in the study. Study design considers the likelihood of bias in the allocation process for observational designs and, for experimental designs, the extent to which assessments of exposure and outcome are likely to be independent. Confounding assesses how far the essential study variables are controlled for during the data analyses and/or in the study design (by stratification or matching). Blinding examines detection and reporting bias, such as whether the researchers, persons providing the intervention, data collectors, and data analysts were aware of the research condition and/or the subjects were aware of the research question(s). Data collection methods are rated on how valid and reliable the tools for primary outcome measures, including distinctions between self-reported data; objective data retrieved by investigators, such as assessment or screening; and extracted data from medical records or vital statistics, are, and withdrawals or dropouts examines the percentage of participants remaining in the study—in other words, who managed to complete the study through the final data collection period (if applicable).

Each of these items, if applicable, was rated as having a low, moderate, or high risk of bias based on the EPHPP quality assessment tool standard guidelines. Subsequently, the overall outcomes from each domain were then translated into a global rating. The global ratings for the studies are as follows; weak-quality (in which two or more factors are rated as weak), moderate-quality (in which one aspect is rated as weak), and strong-quality (in which no characteristics are rated as weak) (42). Study assessments of quality were conducted independently by all authors (KM, SMY, ZI, and ARI). Discussions resolved any discrepancies and queries that arose during the review process.

### Risk of Bias

The authors decided to assess the risk of bias pertaining to the human observational studies done at sewage plants based on three common study biases: information, selection, and confounding biases (43–45). To evaluate the information bias, potential occupational respiratory hazard exposure levels were classified into individual or ecological measurements. Ecological measurement bias might come from environmental assessment tools or monitors placed at the worksites from using job tasks as categories of exposure, improper air-quality assessment conducted by the researchers, and selected worksites as a measure of exposure. Individual measurement bias may be present via a personal sampler measuring instrument on the sewage workers' bodies. The measurement might be distorted by contaminated work clothing, improper monitoring of workers' activities while conducting the assessment, and other sources of exposure at the sewage plants.

The confounders present in the studies were also assessed. An example of confounders would be the association between respiratory hazard exposure and respiratory health effects that might be distorted by smoking. Cigarette smoking indirectly may contribute to the increase in particulate matter 2.5 (PM 2.5) concentration in the air and the development of respiratory illnesses (46). Thus, studies were classified by whether or not they controlled for potentially confounding variables. Last, the authors looked into the possibility of selection bias in all these studies. The selection bias can result from selecting a nonrepresentative sample of the study population. For instance, workers who are exposed to potential occupational respiratory hazards at sewage plants, such as bioaerosols, and experience respiratory symptoms may join the study more often than workers without respiratory symptoms. Studies were classified as to whether they applied a “convenience sample” or “others” for the subject selection. Subject selection was done through a convenience sampling method, such as volunteers being subjected to selection bias. This may result in misleading findings and conclusions.

## Results

### Study Selection

Figure 1 shows a flowchart of the literature search with 5,660 research articles identified through an initial online database search. There were 18 additional studies retrieved from the bibliography search. After the screening process, we removed duplicates and ineligible items and were left with only 26 articles to be included in the systematic review.

### Study Characteristics and Risk of Bias

Tables 1, 2 summarize the study characteristics of the 26 eligible studies. The tables provide information on study designs, study locations, number of participants, risk of bias, and potential respiratory hazards found in each study. Table 1 summarizes 15 observational studies among human subjects, and Table 2 summarizes 11 environmental assessment studies done at sewage plants.

**Table 1:** Human assessment (observational studies) done at the sewage industry included in the systematic review.

**References**	**Study design**	**Study location/subjects**	**Number of subjects**	**Assessments**	**Outcomes**	**Risk of bias**			**Potential hazards**	**Global ratings**
						**Information**	**Confounding**	**Selection**		
Al Batanony et al. (15)	Cross-sectional	Sewage workers at Berket Al-Sabih WWTP, Egypt and Workers at Departments of faculty of Commerce, Menoufiya University, Egypt	86 subjects: 43 exposed and 43 non exposed	Interview, Spirometry examination, 12-lead Electrocardiogram (ECG), Quantitative sandwich enzyme linked immunosorbent assay (ELISA), Polymerase chain reaction (PCR)	Exposed workers had a significant decrease in the mean value of FEV % and PEF %. Exposed workers had significantly higher mean sulf-heamoglobin % as compared to non exposed workers.	I	Controlled (Age and smoking habit)	O	Hydrogen Sulfide	Moderate
Cyprowski et al. (16)	Cross-sectional	Combined STP in Central Poland	78 STP workers	Endotoxin assessment via personal aerosols samplers, Spirometry examination	Sewage sludge treatment workers (SSTW) recorded the highest exposure level to Endotoxin (89.5 EU/m^3^) and inhalable dust (0.24 mg/m^3^). There was a weak positive correlation between the level of inhalable dust and endotoxin concentration (*p* = 0.003, *r* = 0.33). Low levels of endotoxin exposure among workers contributed to a significant impact on declining in FEV1 (*p* = 0.044)	I	Controlled (Inhalable dust exposure and smoking habit)	O	Inhalable dust, Endotoxin	Moderate
Douwes et al. (17)	Cross-sectional	Dutch STP workers	151 STP workers (only 147 returned the questionnaire)	Self-reported health questionnaire, Personal inhalable dust and endotoxin samplers	The upper and lower respiratory symptoms were significantly higher in respondents working 5–10 years (*p* < 0.001) Flu-like symptoms were significantly associated with exposure to sewage (OR = 5.0). No difference in endotoxin and inhalable dust exposure were observed between both plants and between the different seasons. Relatively low endotoxin and inhalable dust exposure among the subjects recorded with a geometric mean of (9.5 EU/m^3^ and 0.3 mg/m^3^) respectively.	I	Controlled (Age and smoking)	C	Endotoxin, Inhalable dust (NS)	Weak
Heldal et al. (18)	Cross-sectional	Eight municipal sewage plants workers in Norway	80 subjects: 36 unexposed and 44 exposed	Self-administered questionnaire, Spirometry examination, Personal Air Sampler Chemi-luminescence Analyzer to measure nitric oxide, Acoustic rhinometry to measure the cross-sectional area and volume of the nasal passage, C-reactive protein blood investigation	Sewage workers handling dry sludge had a higher prevalence of respiratory symptoms and significantly lower FEV1/FVC ratio as compared to non-dry sludge workers (*p* < 0.001). Endotoxin and inhalable dust exposure were significantly higher among sludge treatment workers as compared to plant workers who not involved with sludge treatment (*p* < 0.05). Nitric monoxide exposure among sewage workers was not significant as compared to controls	I	Controlled (Age and smoking)	O	Inhalable dust, Endotoxin, Nitric monoxide (NS)	Moderate
Heldal et al. (19)	Cross-sectional	Eight municipal sewage plants workers in Norway	82 subjects: 44 cases and 38 controls	Self-administered questionnaire, Personal Air Sampler, Blood samples for CC16, SP-A, and SP-D to measure pneumoproteins	Positive correlations between endotoxin and dust concentrations (*rp* = 0.47, *p* < 0.01) and between endotoxin and bacteria concentrations (*rp* = 0.37, *p* < 0.05). Exposed subjects had significantly lower mean concentration of CC16 in serum as compared to the referents (*p* = 0.008)	I	Controlled (Age, sex, atopy, and smoking)	O	Inhalable dust, Endotoxin, Bacteria	Moderate
Heldal et al. (20)	Longitudinal	Sewage workers from four STPs in small rural communities (Steinkjer, Støren, Klæbu, Selbu)	148 subjects: 121 cases and 27 referents (control)	Self-administered questionnaire, Spirometry examination, Personal Air Sampler, Blood samples (to measure ICAM-1, CRP, MIP-1alpha, interluekin-8, CC 16, and surfactant protein D (SP-D)	The highest exposure level of endotoxin was recorded at the sewer net system (342 EU/m^3^) and the lowest exposure level at grease handling with median (13 EU/m^3^). The exposure was significantly associated with the working operation (*p* < 0.05) and season (*p* < 0.05). Only 9% of all H_2_S recording shown a peak at above (10 p.p.m). The job with the most excessive exposure to H_2_S was collecting sewage from cesspools (273 p.p.m.). Sewage plant and sewer workers had a significantly higher prevalence of work-related airway symptoms as compared to referents (33 and 11%, respectively). Noted a significant lower in FEV1% and FVC% among sewage plant and sewer workers as compared to referents. No significant difference in spirometry parameters among sewage workers between the studied sewage plants. There was negative association between ICAM-1 and exposure to H_2_S among sewer net workers (β = −52.6, *r*^2^ = 0.07, *p* < 0.05). There was negative association between FEV1 and Endotoxin exposure among sewer net workers (β = −0.22, *r*^2^ = 0.18, *p* < 0.05).	I	Controlled (Age, smoking, body mass index, and atopy)	O	Endotoxin, Hydrogen Sulfide	Strong
Lee et al. (21)	Cross-sectional	4 Wastewater treatment plant (WWTP), Iowa, United States and 21 water treatment plant (WTP), Iowa, Unites States	147 Subjects: 93 WWTP workers and 54 WTP workers	Self-administered questionnaire, Spirometry examination, Personal Air Sampler of hydrogen sulfide and endotoxin	WWTP workers had a significantly higher prevalence of respiratory symptoms as compared to WTP workers (OR 2.7, 95% CI; 1.1–72.7). The majority of H_2_S samples (95.2%) were less than the threshold limit value (TLV) of 1 p.p.m. 64.5% of the endotoxin sample concentrations exceeded 50 EU/m^3^. WWTP workers suffered from respiratory symptoms known to be associated with hydrogen sulfide exposure. There was no significant association between endotoxin exposure and the development of respiratory symptoms among the WWTP and WTP workers.	I	Controlled (Smoking, use of respirator, and allergy)	O	Endotoxin, Hydrogen Sulfide	Moderate
Melbostad et al. (22)	Longitudi-nal	15 municipal STP in eastern Norway	24 sewage workers	Health symptoms reporting system, Personal sampling pump	No significant correlation between exposure of endotoxin and bacteria (*r*^2^ = 0.00). There was a significant association between exposure to rode-shaped bacteria and the presence of tiredness (*p* < 0.05) and headache symptoms (*p* < 0.05). There was no significant relationship between symptoms and exposure to hydrogen sulfide and endotoxin during a work shift.	I	Did Not Control	O	Bacteria Endotoxin (NS), Hydrogen Sulfide (NS)	Moderate
Richardson (23)	Cross-sectional	Sewer and water treatment workers in Durham and Winston-Salem, North Carolina	223 Subjects: 107 sewer workers and 116 water treatment workers	Self-administered questionnaire, Spirometry examination, Personal Air Sampler of hydrogen sulfide and endotoxin. Job titles were used to categorize the presumed levelof occupational H,S exposure.	Sewer workers appeared to have lower lung function values than water treatment workers. FEV,/FVC values in the smoker and non-smoker sewer workers were statistically significantly lower compared to water treatment workers 11.0 (*p* = 0.03) and −7.8 (*p* = 0.06), respectively. Workers classified as high H_2_S exposure consistently had the lowest observed FEV1/FVC values.	I	Controlled (Age, height, race, and Smoking)	O	Hydrogen Sulfide	Moderate
Rylander (24)	Cross-sectional	Sewage workers in eight sewage treatment plants in four municipalities in the south of Sweden	69 subjects: 34 sewage workers (cases) and 35 controls	Interviewed administer questionnaire, Spirometry examination, Stationary air sampler (Air sampling filters)	The vicinity of sludge handling and during cleaning were recorded as the highest amount of airborne endotoxin range 2–32 170 ng/m^3^). However, the amount of endotoxin presence at sewage treatment plants was not exceeded the normal background value. Sewage workers had a higher prevalence of airway symptoms as compared to controls after adjusted by smoking status. The Methacholine induced decrease in FEV1 was significantly higher among sewage workers compared to controls −6.2 (5.8) and −4.8 (3.9), respectively	IE	Controlled (Smoking and subjects who had past exposure in dusty industry)	O	Endotoxin	Moderate
Saad et al. (25)	Cross-sectional	Old STP located at the South-west of Cairo city, New STP located at the South-west of Cairo city	61 newSTP workers and 46 old STP workers and compared with 40 non-exposed controls	Self-administered questionnaire, Spirometry examination, Stationary air sampler using calibrated vacuum pump, dry gas meter, and large glass bubblers	Sewage workers working at the new plant had a significantly higher prevalence of developing acute bronchitis (*p* < 0.05) compared to controls. Both plants (new and old) had significantly lower mean FVC% of predicted sewage workers as compared to controls. Old plants sewage workers had significantly lower FEV1% of predicted and the FEV1/FVC% value compared to controls. The highest concentration of H_2_S and NH_3_ were found at screening tanks of new plants. While the aeration tanks of the old plant recorded the highest concentration of NO_2_. Exposure to hazardous chemical pollutions (H_2_S, NH_3_, NO_2_, SO_2_, and HCHO) among the sewage workers caused a significant effect on pulmonary function.	IE	Controlled (Smoking)	O	Hydrogen sulfide, Nitrogen dioxide, Ammonia, Sulfur dioxide, Formalde-hyde	Moderate
Smit et al. (26)	Longitudi-nal	Twenty seven Dutch Water Boards workers	468 subjects: 97 office workers, 371 operators and maintenance workers	Self-administered adapted version of questionnaire, Personal endotoxin exposure sampler	Personal 8 h endotoxin exposure was low with a geometric mean of (27 EU/m^3^). Wastewater workers had a higher prevalence of having cough symptoms compared to the general population (*p* < 0.001). Wastewater workers who were exposed to the highest level of endotoxin (>200 EU/m^3^) had a higher prevalence ratio in having flu-like and upper respiratory symptoms [PR: 2.02 (95% CI: 0.83–4.88)] and [PR: 1.79 (95% CI: 0.84–3.84)].	I	Controlled (Age, gender, and smoking habits)	C	Endotoxin	Moderate
Tabrizi et al. (30)	Cross-sectional	Wastewater and garbage workers in Canton, Zurich	778 Subjects: 395 controls and383 cases (316 wastewater workers 67 garbage workers)	Self-administered questionnaire, Spirometry examination, Personal Air Sampler of Endotoxin, ELISA	The mean exposure levels to endotoxins in both plants were below 100 EU/m^3^. Special tasks of wastewater workers caused higher endotoxin exposure with a mean of 98.6 EU/m^3^ (1.4–497). There was no significant difference in the level of FEV1/FVC between the 3 groups (*p* > 0.1). No clinically relevant correlation between spirometry results and SP-D concentrations appeared.	I	Controlled (Age, gender, previous or current work as a farmer, and smoking habits)	O	Endotoxin	Moderate
Thorn (28)	Cross-sectional	five municipal STP in western Sweden	114 Subjects: 59 operatives workers (cases) and 55 controls	Self-administered questionnaire, Spirometry examination, Personal and Stationary Air Samplers, ELISA, PCR, Nasal Lavage	The prevalence of presence respiratory symptoms among cases was higher than cases and most likely due to endotoxin exposure. No significant difference in pulmonary function values between the cases and controls. Most of the H_2_S reading was low (<1 p.p.m). the highest recorded at 6 p.p.m	*IE*	Did not control	O	Endotoxin, Hydrogen Sulfide	Weak
Tschopp et al. (29)	Cross-sectional	Wastewater and garbage workers in Canton, Zurich	603 Subjects: 304 controls, 299 cases (247 wastewater and 52 garbage workers)	Interviewed and Self-administered questionnaire, Spirometry examination, Personal and stationary Air Sampler of Endotoxin, ELISA	The highest peak exposure of endotoxin up to 500 EU/m^3^ was identified among wastewater workers. No significant association between exposure to endotoxin and respiratory symptoms development, spirometry value, and specific protein concentration throughout the study between the subgroups. The effect of occupational exposure toward endotoxin was not significant	I	Controlled (Obesity, previous or current work as a farmer, and smoking habits)	O	Endotoxin	Moderate

*E, Ecological; I, Individual; C, Convenience; O, Others; NS, Not Significance*.

*OR, Odds ratio; PR, Prevalence ratio; H_2_S, Hydrogen sulfide; NO_2_, Nitrogen dioxide; NH_3_, Ammonia; SO_2_, Sulfur dioxide; HCHO, Formaldehyde; PM 2.5, Particulate matter 2.5; VOC, Volatile organic compound; FEV1, Forced expiratory volume in 1s; FVC, Forced vital capacity; PEF%, Peak expiratory flow percent of predicted value; FEV%, Forced expiratory volume percent of predicted value; WWTP, Wastewater treatment plant; STP, Sewage treatment plant; SSTW, Sewage sludge treatment workers; COPD, Chronic obstructive pulmonary disease; ELISA, Enzyme linked immunosorbent assay; PCR, Polymerase chain reaction; SP-D, Surfactant protein D; SP-A, Surfactant protein D; CRP, C-reactive protein; ICAM, Intercellular adhesion molecule; MIP, Int macrophage inflammatory protein; CC16, Club cell protein 16*.

**Table 2:** Environmental exposure assessment studies done at the sewage industry included in the systematic review.

**References**	**Study location**	**Number of samples**	**Assessments**	**Outcomes**	**Potential hazards**	**Global ratings**
Austigard et al. (30)	Two areas:Two WWTP in the cities and three WWTP in the rural area, middle of Norway	93 sample measurements,149 sewage workers were selected and divided into four group: (i) Big plants (ii) Pumps station (iii) Sewer network (iv) Collecting sewage from cesspools	Personal air sampling for H_2_S	8 out of 93 samples (9%) of H_2_S measurements had recorded peaks above 10 p.p.m. Workers working at collecting sewage from cesspool station had the highest level of exposure to H2S. The determinants that could affect the H2S index were job type, season, plant location, and degree of flushing.	H_2_S	Strong
Carducci et al. (31)	WWTP in Italy	Numbers of samples not mentioned. Four locations of study: (i) Sewage effluent (ii) Biological oxidation tank (iii) Sludge treatment (iv) Side entrance manhole	Secondary data, Stationary air sampling for bioaerosol using impactor sampler loaded with Rodac plates, QMRA	Workers who worked in the sewage effluent and biological oxidation tank had a higher probability of illness in relation to the exposure of the bioaerosols with very good reliability (*r*^2^= 0.92). There were estimated higher quantitative microbial average risk exposure in sewage influent and biological oxidation tanks (15.64 and 12.73% for an exposure of 3 min). Human adenovirus concentration is a predominant factor in the estimated risk.	Bioaerosol (Human Adenovirus)	Moderate
Cyprowoski et al. (32)	WWTP in Poland	Six sampling points of both sewage and sludge station, at 10 different workplaces in WWTP	Stationary air sampling using 6-stage Andersen impactor, Petri plates containing Schaedler agar. Samples collected at single repetition in July 2014 and February 2015.	The average concentration of anaerobic bacteria in the sewage samples was 5.49 × 104 CFU/m^3^ (GSD = 85.4) and in sludge 1.42 × 106 CFU/g (GSD = 5.1). Winter at the bar screens recorded the highest bacterial contamination (4.06 × 103 CFU/m^3^). 16 bacterial species were determined, from which the predominant strains belonged to 5 genera which were; *Actinomyces, Bifidobacterium, Clostridium, Propionibacterium*, and *Peptostreptococcus* genera. Mechanical treatment processes caused a substantial emission of anaerobic bacteria into the air. No significant difference in bacterial biota in the air between sewage and sludge station.	Bioaerosol (Airborne Anaerobic Bacteria)	Strong
Gotkowska et al. (33)	Domestic WWTP, Ostro'da in the north-east of Poland	288 air samples were taken: Contorls = 24, Mechanical treatment = 96, Biological treatment = 72, and surroundings = 96 12 sampling sites: (i) 1 control site (ii) 7 sites located in the WWTP (grate chamber, grit chamber, retention chamber, preliminary settling tank, pre-denitrification tank, nitrification, and denitrification tanks, secondary sedimentation tank (iii) 4 sites located outside the WWTP (at the fence of the plant, and 50, 100, and 200 min from the fence)	Stationary air sampling via impact sampler surface air system MAS-100 Eco Merck with 400 holes, Petri plates containing agar. Air sampling taken in two annual cycles (in 2005 and 2006) and in four different seasons.	Overall, 25 species of microorganisms were identified. Higher numbers of HPC bacteria in air samples were observed in summer, fungi in autumn. The mechanical sewage treatment produced significant emission of microorganisms to the air (the grate chamber, the grit chamber, the preliminary settling tank). While biological sewage treatment equipped with a fine bubble aeration system produced only a small amount of bioaerosols.	Bioaerosol (Airborne Bacteria)	Strong
Oppliger et al. (34)	11WWTP in Canton of Zurich, Switzerland.	22 air samples from 11 sewage treatment plant (indoor and outdoor)	Personal air sampling using polycarbonate filters, Stationary air sampling, Agar plates. Air sampling taken in two season which were winter and summer. Four hours sampling in two reading per day.	The fungi concentration was significantly higher in summer as compared to the winter season (2,331 ± 858 vs. 329 ± 95 CFU/m^3^). Particle grids for incoming water in the enclosed areas had a significantly higher bacteria concentration as compared to the unenclosed areas near the aeration basins in both winter and summer seasons. All bioaerosols were frequently above the recommended values of occupational exposure. The sewage workers who needed to conduct special tasks such as cleaning tanks were exposed to a very high levels of endotoxins (up to 500 EU/m^3^) compared to routine work. The most predominant species of bacteria found were from Pseudomonadaceae and the Enterobacteriaceae family.	Bioaerosols (Airborne Bacteria)	Moderate
Shiota et al. (35)	Sewage sludge incinerators (SSI) in Japan	5 sampling points: (i) 2 dry Electrostatic precipitators (EP) (ii) 1 wet EP (iii) 1 bag filter (iv) 1 ceramic filter	Andersen stack samplers to measure particulate matter	The average PM 2.5 concentration was 0.00014–4.8 mg/Nm^3^. Annually, an estimated about 0.96–8.9 tons of PM 2.5 was emitted into the air. The highest contribution of PM 2.5 emission was at dry EP (77–99% of total emission).	PM 2.5	Moderate
Spaan et al. (36)	43 Dutch sewage treatment plants	647 air samples were taken: (i) 470 full shift personal (ii) 123 tasked-based (iii) 54 stationary measurements	3 occupational exposure assessments methods applied: Personal, Stationary, Task-Based	There was moderate to low endotoxin exposure among the sewage treatment plant workers. The highest exposure recorded was through task-based method and followed by stationary and the least was personal sampling with an overall geometric mean of 64, 33, and 27 EU/m^3^, respectively. No significant determinants of day to day exposure variability within and between the workers. The highest endotoxin levels recorded via stationary measurements were found in the front end of the process has, whereas the highest dust concentrations were found during sludge dewatering. The highest amounts of total bacteria counts found in the sludge dewatering system were gram-positive bacteria and fungi levels were higher as compared to gram-negative bacteria levels.	Bioaerosol (Endotoxin and Bacteria)	Moderate
Upadhyay et al. (37)	WWTPs in the Phoenix (AZ) metropolitan area	24 air samples were taken 2 main locations: (i) Covered area basin (CAB) (ii) Open area basin (OAB)	Field sampling methods: PM sampling system, annular denuders to measure ammonia concentration, ChemVol impactor to measure organic trace. Air sampling taken within 1 year duration of interval.	The concentrations of PM 2.5, PM10, and NH3 at the aeration basins were similar and within urban ranges. PM 2.5concentration at the CAB basin was the highest compared to other samples. NH3 concentrations were highest at the CAB facility (13 to 72 mg/m^3^) above the aeration basin. However, there was no significant difference between NH3 concentration at both location CAB and OAB.	PM 10, PM 2.5, Ammonia	Strong
Yang et al. (38)	WWTP at Harbin City in northern China	Numbers of samples not mentioned	Stationary air and water sampling for VOCs. Four sampling seasons were monitored.	Three aromatic hydrocarbons, notably benzene, were more readily released from the wastewater into the atmosphere. The primary clarifier of the WWTP had the highest VOC concentrations during summer. VOC especially polyaromatic hydrocarbon expected had long-distance traveling to the surrounding area as was observed at locations far away from the WWTP.	VOC	Strong
Yang et al. (39)	WWTP located in Beijing, China.	7 sites: (i) Coarse screen (CS) (ii) Aerated grit chamber (AGC) (iii) Primary settling tank (PST) (iv) Anaerobic tank (AnT) (v) Aeration tank (AeT) (vi) Secondary settling tank (SST) (vii) Sludge dewatering house (SDH)	Stationary air sampling for bioaerosol, Total suspended particles (TSP) sampling, Nutrient agar to measure airborne bacteria, Ion Chromatography System to measure anions concentration. Three sampling for each season; winter spring and summer.	There were positive correlations between sites and bacterial concentrations were observed in winter, spring, and summer (ANOVA 1, *p* < 0.001). The highest emission level of airborne bacteria recorded at treatment stages of CS, AGC, PST, AnT, and AeT ranged from 257 to 4,878 CFU/m^3^. The concentration of airborne bacteria was significantly lower at the external sites of WWTP as compared to internal sites of WWTP (*p* < 0.001). Assume to be due to wind dilution and dispersion effect. The main anions were detected; chloride, nitric oxide, and sulfur dioxide. Inhalation risk magnitude is higher among the WWTP workers as compared to skin contact risk especially at worksites with high levels of airborne bacteria, such as AGC, CS, and AeT. Inhalation risks of airborne bacteria in summer were higher than those in the other two seasons.	Bioaerosol (Airborne bacteria), NO, SO_2_, Cl	Srong
Prazmo et al. (40)	Sewage treatment plant located in eastern Poland	12 sites: (i) 9 at Sewage treatment Plant (ii) 2 at City pump station (iii) 1 sewer duct	Stationary air sampling for airborne endotoxin Agar media. Five double air samples taken from each site.	The sampling site was a significant factor in determining quantities of airborne microorganisms (*p* < 0.001). The initial phase of STP processes which were clearing, primary sedimentation, aeration had 2–3 times higher airborne microorganism loads compared to those at final phase STP processes which were secondary sedimentation and sludge dewatering. The majority of bacteria found in eight sites were corynebacteria. Three-quarter of the samples shown positive fungi aerosols. The airborne endotoxin concentration in the plant was low and within the range of 0.104–5.2 ng/m^3^. The concentrations of microorganisms and endotoxin were not significantly correlated (*p* > 0.05).	Bioaerosol (Airborne endotoxin), Bacteria, Fungi	Weak

*H_2_S, Hydrogen sulfide; NO, Nitrogen oxide; SO_2_, Sulfur dioxide; Cl, Chloride; PM 2.5, Particulate matter 2.5; VOC, Volatile organic compound; WWTP, Wastewater treatment plant; STP, Sewage treatment plant; QMRA, Quantitative microbial risk assessment; HPC, Heterotrophic bacteria; CFU, Colony forming unit; GSD, Geometric standard deviation*.

### Results of Human Observational Studies

The quality of the human observational studies included in this review was assessed with most studies (12 out of 15) having a moderate-quality rating, two rated as weak, and one rated as strong. Quality assessment results can be seen in Table 3. Regarding the sewage workers' exposure to potential occupational respiratory hazards found at sewage plants (see **Table 5**), most of the articles (12 out of 15) studied “endotoxin” exposure. Seven out of 15 articles studied the effects of hydrogen sulfide (H_2_S) exposure among sewage workers. Next, four articles focused on inhalable dust as a potential respiratory hazard. However, two articles studied the presence of nitric oxide (NO), nitrogen dioxide (NO_2_), ammonia (NH_3_), sulfur dioxide (SO_2_), and formaldehyde. Concerning the implemented study design, most were conducted cross-sectionally (12 out of 15), and three studies implemented a longitudinal study design.

**Table 3:** Quality assessment results against the effective public health practice project quality assessment tool for human observational studies.

**References**	**Selection bias**	**Design**	**Confounders**	**Blinding**	**Data collection method**	**Withdrawals/dropouts**	**Global ratings**
Al Batanony et al. (15)	Moderate	Weak	Moderate	Moderate	Strong	Strong	Moderate
Cyprowski et al. (16)	Moderate	Weak	Moderate	Moderate	Strong	Strong	Moderate
Douwes et al. (17)	Moderate	Weak	Weak	Moderate	Strong	Strong	Weak
Heldal et al. (18)	Strong	Weak	Moderate	Strong	Strong	Strong	Moderate
Heldal et al. (19)	Strong	Weak	Strong	Strong	Strong	Strong	Moderate
Heldal et al. (20)	Strong	Moderate	Strong	Moderate	Strong	Strong	Strong
Lee et al. (21)	Strong	Weak	Strong	Strong	Strong	Moderate	Moderate
Melbostad et al. (22)	Moderate	Moderate	Weak	Strong	Strong	Strong	Moderate
Richardson (23)	Moderate	Weak	Moderate	Strong	Moderate	Moderate	Moderate
Rylander (24)	Strong	Weak	Strong	Strong	Strong	Strong	Moderate
Saad et al. (25)	Strong	Weak	Moderate	Strong	Strong	Strong	Moderate
Smit et al. (26)	Moderate	Weak	Strong	Strong	Strong	Strong	Moderate
Tabrizi et al. (27)	Moderate	Weak	Strong	Moderate	Strong	Strong	Moderate
Thorn (28)	Strong	Weak	Weak	Strong	Moderate	Strong	Weak
Tschopp et al. (29)	Moderate	Weak	Strong	Moderate	Strong	Strong	Moderate

#### H_2_S Exposure Among Sewage Workers

Al Batony et al. (15) conducted a moderate-quality cross-sectional study among 86 subjects to determine the effects of H_2_S exposure among sewage workers at a wastewater treatment plant (WWTP) in Egypt. They found the exposed workers prone to respiratory symptoms (wheezing and asthma) (*p* < 0.05) and a significant decrease in the mean value of FEV% and PEF% from the spirometry assessment (*p* < 0.05). They also found that the exposed workers had a higher mean of sulfhemoglobin percentage than nonexposed workers (*p* < 0.001). The same findings were discovered in a moderate-quality study done in 1995 by Richardson (23); the author found that the sewer workers had lower FEV1/FVC values than water treatment workers 11.0 (*p* = 0.03) and −7.8 (*p* = 0.06), respectively, after adjusting for smoking habits. These significant findings were consistent with the higher exposure of H_2_S recorded among sewer workers when compared to water treatment workers. Both of these studies emphasize H_2_S as the potential respiratory hazard found in sewage plants and significantly affecting the workers' respiratory health.

#### H_2_S and Other Chemical Air Pollutant Exposure Among Sewage Workers

We found five articles studying more than one substance in addition to H_2_S. One moderate-quality study looked into H_2_S and other chemicals, such as NO_2_, SO_2_, NH_3_, and formaldehyde (25). The study was conducted in the old and new STPs in Cairo city and measured the effects of those exposures on workers' respiratory health. They established significantly lower mean values of FVC% of predicted exposure to all studied respiratory hazards among both groups of STP workers than controls. The aeration tank workers at the new plant and screening tank workers at the old plant recorded the lowest mean values of FVC% predicted 57.9 ± 10.7 and 54.0 ± 13.9, respectively. Different STPs used different techniques and processes, and this may produce various sources of hazards.

#### H_2_S and Endotoxin Exposure Among Sewage Workers

Meanwhile, the other four articles studied the effect of H_2_S and endotoxin exposure among sewage workers. The latest study was conducted by Heldal et al. (20); this is the only strong-quality study found in this review. It is a longitudinal study done among 148 sewage workers in Sweden. In this study, the authors planned to take samples over a year, including four seasons. The authors found that only 9% of all H_2_S samplings recorded a peak above 10 ppm in which the threshold limit value (TLV) is 1 ppm, and the highest exposure was among sewage workers who do the job of collecting sewage from a cesspool (273 ppm). Next, there was a significant negative association between exposure of endotoxins and FEV1% value among sewer net workers, which means that the higher endotoxin exposure among workers produced a lower FEV1% value.

Additionally, there was a significantly lower FEV1 and FVC% among sewage workers than referents after adjusting for age, smoking, and BMI. The endotoxin and H_2_S exposure among the subjects were confirmed to be associated with seasonal variation and working operation. A weak-quality study done among sewage workers in Sweden reported that endotoxin exposure might contribute to developing respiratory symptoms among the subjects (28). However, this study's findings were questionable as this study did not control for any confounders during the selection of subjects or in the data analysis.

On the other hand, two moderate-quality studies done by Lee et al. (21) and Melbostad et al. (22) did not include or discuss seasonal variation effects. Lee et al. (21) found most H_2_S samples collected in the study were less than TLV of 1 ppm. However, it was found that a higher prevalence of WWTP workers had respiratory symptoms compared with WTP workers, and this was associated with H_2_S exposure. In comparison, exposure to endotoxins was not found to be related to the development of respiratory symptoms in both worker groups. However, more than half of the study's air samples exceeded endotoxin concentration (>50 Eu/m^3^). Paradoxically, there was no association between H_2_S and endotoxin exposure among 24 sewage workers during the work shift in eastern Norway (22). However, the authors found a significant association between exposure to bioaerosol (bacteria) and the presence of tiredness symptoms (*p* < 0.05).

#### Endotoxin Exposure Among Sewage Workers

Next, four moderate-quality articles looked into endotoxin exposure and its effect on sewage workers' respiratory health. Two out of the four studies show that there is a significant association between exposure to endotoxins and respiratory health among sewage workers [i.e., development of respiratory symptoms (26) and deterioration of pulmonary function (24)]. Rylander et al. (24) conducted a study among 69 sewage workers in south Sweden. They establish endotoxins at the sewage plants, and the highest concentration recorded at the sludge handling and cleaning section ranged from 3.8 to 32 170 ng/m^3^. The endotoxins were confirmed to cause airway inflammation.

Another study in the Netherlands at 27 sewage plants reveals a low geometric mean of endotoxin exposure (27 Eu/m^3^) found in the study. Wastewater workers exposed to a higher level of endotoxins (>200 Eu/m^3^) had a higher prevalence ratio in developing flu-like and respiratory symptoms. In contrast, another two articles prove no association between the level of endotoxin exposure and the presence of adverse respiratory health effects (27, 29). Both of these studies were conducted among wastewater and garbage workers in Zurich. The authors from both studies found that endotoxin exposure in both plants was low (<100 Eu/mg^3^). Also, endotoxin exposure in both groups of workers' spirometry values was not significant throughout the subgroups after adjusted for smoking and obesity.

#### Inhalable Dust Exposure Among Sewage Workers

Inhalable dust is also a potential respiratory hazard found at sewage plants and is shown to be associated with the development of respiratory symptoms and decreased lung function. These facts are proven in two moderate-quality studies by Heldal et al. (18, 19). The authors conducted a cross-sectional study at eight municipal plants in Norway. They established there was endotoxin and inhalable dust exposure among the workers. The exposures were significantly higher among sludge treatment workers as compared with workers not involved in sludge treatment. In addition, sludge workers tend to have a higher prevalence of developing respiratory symptoms and a significant reduction in spirometry values, especially the FEV1/FVC ratio (*p* < 0.001). There was also a significant positive correlation between endotoxin and dust concentration at the sewage plant (*rp* = 0.47, *p* < 0.01).

However, the other two studies show contradictory findings on the presence of inhalable dust exposure and its effect on sewage workers' respiratory health. First Cyprowski et al. (16) find a weak positive correlation between endotoxin and inhalable dust (*r* = 0.33, *p* = 0.003). The inhalable dust exposure among the workers was low and below the exposure limit. It is shown that the FEV1 significantly declined among the workers who had the highest endotoxin exposure, but it was independent of inhalable dust and smoking habits. Second a weak-quality study conducted among 151 STP workers shows no significant difference in exposure of endotoxins and inhalable dust throughout the four seasons with a geometric mean of 9.5 Eu/m^3^ and 0.3 mg/m^3^, respectively (17). The endotoxin and dust exposure among the workers were unable to explain the development of flu-like symptoms. It could be affected by other associated factors as well. However, this study was classified as weak quality due to the weak study design and was not properly controlled by the confounders.

### Results of Environmental Assessment Studies

The quality of the environmental assessment studies included in this systematic review were assessed. Most studies (6 out of 11) have a strong-quality rating; four are rated as moderate, and one rated as weak (see Table 4). Regarding the method of air sampling assessment, most of the articles (7 out of 11) utilized stationary air sampling to determine and measure the potential hazard concentration at the sewage plants. Only one study used an individual or personal air sampling technique. In contrast, two studies utilized both personal and stationary air sampling techniques. On top of that, task-based air sampling and personal and stationary sampling were used in a study done by Spaan et al. (36).

**Table 4:** Quality assessment results against the effective public health practice project quality assessment tool for environmental assessment studies.

**References**	**Selection bias**	**Design**	**Confounders**	**Blinding**	**Data collection method**	**Withdrawals/Dropouts**	**Global ratings**
Austigard et al. (30)	Strong	Moderate	Strong	Moderate	Strong	Strong	Strong
Carducci et al. (31)	Moderate	Moderate	Weak	Moderate	Moderate	Strong	Moderate
Cyprowoski et al. (32)	Strong	Moderate	Moderate	Moderate	Strong	Strong	Strong
Gotkowska et al. (33)	Moderate	Moderate	Moderate	Moderate	Moderate	Moderate	Strong
Oppliger et al. (34)	Strong	Moderate	Weak	Moderate	Strong	Strong	Moderate
Shiota et al. (35)	Strong	Moderate	Weak	Moderate	Strong	Strong	Moderate
Spaan et al. (36)	Strong	Moderate	Weak	Strong	Strong	Strong	Moderate
Upadhyay et al. (37)	Moderate	Moderate	Moderate	Moderate	Moderate	Strong	Strong
Yang et al. (38)	Strong	Moderate	Moderate	Strong	Strong	Strong	Strong
Yang et al. (39)	Strong	Moderate	Moderate	Moderate	Strong	Strong	Strong
Prazmo et al. (40)	Moderate	Weak	Weak	Moderate	Strong	Strong	Weak

The majority of the articles (5 out of 11) measured bioaerosol or endotoxin concentration present at sewage plants (see Table 5). These include six articles that measured bacteria concentration, and the other article studied virus concentration at sewage plants. Next, there were two articles reviewed on the presence of PM 2.5, one article on H_2_S, one article on volatile organic compounds (VOC), and two articles on several chemicals (e.g., NO, chloride, NH_3_, and SO_2_) concentration at sewage treatment plants.

**Table 5:** Summary of occupational respiratory hazards studies included in the systematic review.

**Studied potential occupational hazards**	**Articles references**
**Human observational studies**	
(1) Endotoxin	(16–22, 24, 26–29)
(2) Hydrogen sulfide	(15, 20–23, 25, 28)
(3) Inhalable dust	(16–19)
(4) Other chemicals Nitric monoxide (NO), Nitric dioxide (NO_2_), Ammonia (NH_3_), Sulfur dioxide (SO_2_), Formaldehyde	(18, 25)
**Environmental assessment studies**	
(1) Bioaerosols or endotoxin	(31–34, 36, 39, 40)
(2) Particulate matter	(35, 37)
(3) Hydrogen sulfide	(30)
(4) volatile organic compound	(38)
(5) Other chemicals Nitric monoxide (NO), Chloride (Cl), Sulfur dioxide (SO_2_), Ammonia (NH_3_)	(37, 39)

#### H_2_S Concentration in the Air

A strong-quality study was conducted by Austigard et al. (30) at 56 WWTPs in the middle of Norway. The authors collected 93 personal air samples over 1 year to measure the exposure of H_2_S among the sewage workers and determine the effect of seasonal variation on the exposure. They found only 1 in 10 (9%) of H_2_S measurements recorded were above TLV level (>10 ppm). In this study, the authors calculated the H_2_S index used to evaluate the exposure and its relation to health effects. The workers who did the job of collecting sewage at the cesspool obtained the highest H_2_S index. Subsequently, the study suggests that the exposure of H_2_S among sewage workers could be affected by several determinants: job type, seasonal variation, location of the plant, and degree of flushing.

#### PM 2.5 Concentration in the Air

Two articles measured PM 2.5 emission at sewage plants. The latest study was conducted by Shiota et al. (35) in 2015. Anderson stack filters were utilized and located at five sewage sludge incinerators in Japan to measure PM 2.5 emission mass concentration. It was found that the PM 2.5 emission was low and close to the environmental standard (35 μg/m^3^ daily). The SSI using the dry electrostatic precipitator method recorded the highest contribution emission of PM 2.5. Next, 24 stationary air samples were taken from a WWTP study in the United States (37). In this strong-quality study, it was found that the emission of PM 2.5, PM 10, and NH_3_ at both types of aeration basin (open and closed system) were within turban range. There were no significant differences found in the emission of those three studied substances in the air between open and closed aeration basins.

#### Bioaerosols or Endotoxin Concentration in the Air

It appears that the studies done so far concerning environmental air assessment at sewage plants are focused on bioaerosols and endotoxins, which include bacteria, viruses, and fungi. One moderate-quality study was conducted at a WWTP in Italy using a stationary air sampling method to quantify the quantitative microbial risk assessment (QMRA) for the human adenovirus (HAdv) among sewage workers (31). The QMRA is useful in assessing health risks at the individual level. The authors found that sewage workers who worked at sewage influent and biological oxidation tanks had a higher risk of HAdv exposure compared with sludge treatment and side entrance manholes with good reliability results (*r*^2^ = 0.92). In addition, sensitivity analysis was conducted and HAdv concentration was found to be a predominant factor to be included in the QMRA.

Regarding the airborne bacteria concentration at sewage plants, a strong-quality study conducted by Cyprowoski et al. (32) tried to assess the exposure to anaerobic bacteria released into the air at the WWTP in Poland. They collected 12 samples for 6 sampling points via stationary air samplers in a year. They found that the anaerobic bacteria widely presented in the air at WWTP workplaces and mechanical treatment processes caused a significant release of anaerobic bacteria emission into the air (*p* < 0.05). Next, there were 16 bacterial species identified, but there were no significant differences in the bacteria's microbiota across the samples taken. There was also no difference in anaerobic bacteria emission between the studied seasons.

However, this finding contradicts a strong-quality study done by Gotkowska et al. (33) that was conducted in the same country but at different WWTP locations. This study found a significant difference in bacteria concentration presented in the air, depending on the sampling season. There was a negative correlation between the number of staphylococci and air humidity (*r* = −0.286, *p* < 0.05) as the air humidity was significantly varied between the seasons. It identified about 25 species of microorganisms in the WWTP air. Again, the mechanical sewage treatment produced substantial emissions of microorganisms into the air. However, it is shown that the number of microorganisms emitted was low if the process utilized fine bubble aeration.

Later, a moderate-quality study conducted by Oplinger et al. (34) used both personal and stationary air sampling and measured indoor and outdoor air at an STP. They found all bioaerosol concentrations to be above the recommended allowable limit of occupational exposure and varied with job tasks. Besides that, the enclosed areas' sewage processes had higher bacteria concentrations compared with the unenclosed areas. In contrast, Prazmo et al. (40) discovered that the airborne endotoxin concentration at the STPs located in eastern Poland was low and within the range of 0.1–5.2 ng/m^3^. On top of that, fungi were identified in most of the samples along with bacteria. There was no significant correlation between microorganisms and endotoxin concentration. However, the findings were questionable as this study did not control any confounders, such as seasonal variation that might affect the concentration of airborne endotoxins at the STP.

Inhalable dust also was found to be present at an STP (36). For personal exposure, mechanics and sludge workers were exposed to a higher concentration of inhalable dust although, for stationary air assessment, the highest dust concentrations were found during the sludge dewatering process. The effect of climate variability over inhalable dust concentration was only explained in a small amount (1–7%). Overall, the inhalable dust and endotoxin exposure levels in Dutch STP were relatively low.

#### Others Hazardous Substance Concentrations in the Air

A strong-quality study conducted by Yang et al. (39) established hazardous substances other than endotoxins in the air at a WWTP in China. The authors were able to detect three major anions: nitric oxide, sulfur dioxide, and chloride. The anions can mostly be found in aerated grit chambers and anaerobic tanks. Regarding the endotoxin concentration, it was significantly higher at internal sites of the WWTP than external sites of WWTP, which heightens the inhalation risk magnitude among WWTP workers toward the microorganisms. Furthermore, the inhalation risk recorded was highest during summer compared with other seasons.

#### VOC Concentration in the Air

A study measured VOC concentrations in the atmosphere at the WWTP in China and surrounding areas (38). In this strong-quality study, it was found that, during summer, the VOC concentration recorded the highest reading. Interestingly, VOC not only can be found in the WWTP atmosphere, but can also travel to the surrounding area ~4 km in radius. The primary clarifier site had the highest VOC concentration during summer. Among the VOC substances found in the study, benzene is the most readily released into the atmosphere.

## Discussion

This systematic review found that studies were done among human subjects in determining the association between potential respiratory hazard exposure and respiratory health among sewage workers, and they were diverse in design and sample sizes. It is difficult to draw solid conclusions due to the diversity in methodological and multiple exposures that could contribute to the respiratory health effects at the same time, such as endotoxins and chemicals. Also, some studies did not control the confounding factors and sometimes had incomplete control. Potential confounders found were age, smoking habit, obesity, gender, use of respirators, and previous history working in the dust industry. Also, self-selection bias by chance might produce an association of respiratory hazards with poor respiratory health outcomes.

Most of the studies utilized a set of questionnaires to assess the presence or development of respiratory symptoms. Spirometry was used to measure pulmonary function. Next, for exposure assessment, the researchers preferred to use personal rather than stationary air sampling to measure the subjects' respiratory hazard exposure. Some studies were conducted extensively using invasive methods, such as taking blood samples, for instance, for polymerase chain reaction (PCR) and enzyme-linked immunosorbent assay (ELISA), to determine the inflammation reaction in the subject's body.

From this review, it is reported that there are plenty of respiratory hazards that can be found at sewage plants. Thus, sewage workers are exposed to various hazards, including bioaerosols, chemicals, or both. The most studied risks were bioaerosols, which include endotoxins, bacteria, and fungi. Most of the studies report that endotoxin exposure could produce significant respiratory health effects among sewage workers in developing respiratory symptoms and a reduction in pulmonary function. The endotoxin concentration in the air differed between different worksites and processes. It is revealed that higher concentrations were identified at the sewage sludge treatment area.

However, a small number of studies found exposure to endotoxins or bioaerosols among sewage workers is not significantly associated with the development of adverse respiratory health effects (22, 27, 29). One of the reasons could be the lower geometric mean of endotoxin exposure among the sewage workers reported in those studies. Therefore, the sewage workers did not have a significant dose of exposure to produce negative respiratory health effects. On top of that, it is reported that the healthy worker effect phenomenon found during the study might be caused by selection bias among the subjects involved in the study (29).

H_2_S was also found to be a potential respiratory hazard among sewage workers. Exposure to H_2_S may cause deterioration in workers' pulmonary function and the development of respiratory symptoms. An invasive study approached using mean sulfhemoglobin% to determine the magnitude of H_2_S exposure. It was found to be significantly higher among exposed workers compared to nonexposed workers. Even though several studies report that H_2_S concentration in the air was moderate or lower than TLV of 1 ppm, the studies managed to find a significant association between H_2_S exposure and the presence of respiratory symptoms among WWTP workers. Usually, sewage workers were more exposed to the incidental H_2_S peak type of exposure than constant exposure. H_2_S was also found to cause negative respiratory effects and cause central nervous system–related symptoms, such as tiredness and concentration difficulties among the sewage workers (47).

There are also studies other than at sewage plants that prove the significant association between H_2_S exposure and adverse respiratory health effects. These studies were conducted at hog operations, housing nearby hog manure lagoons, and in the oil and refineries industries (48–50). However, a study done by Melbostad et al. (22), by contrast, did not find any significant relationship between H_2_S exposure and the presence of respiratory symptoms. This finding parallels other studies done among pulp mill workers by Jappinen et al. (51). One of the reasons behind this was the duration of exposure among the workers. This means that workers exposed for a shorter duration of time to H_2_S may not develop respiratory symptoms or reduce lung function. Another study conducted among 1,204 participants in a community based at the geothermal field in New Zealand had similar findings (52). The authors found that there was no significant association between long-term exposure of ambient H_2_S concentration and lung function decrement or increased risk of chronic obstructive pulmonary disease (COPD) or asthma.

Furthermore, the H_2_S exposure may benefit the lung function as it promotes airway smooth muscle relaxation. However, the authors realized that the findings could be affected by selection bias and exposure misclassification effects. Even though both of these studies were not conducted in the sewage plants, the evidence on H_2_S exposure could not give rise to adverse respiratory effects should it be taken into consideration. The toxic effects of H_2_S are characteristically dose-related, and its impact depends on the frequency and duration of exposure of the individual (53).

Inhalable dust is one of the potential hazards that can be inhaled easily by sewage workers if no proper personal protective equipment (PPE) is applied. Most of the researchers usually studied both inhalable dust and airborne bacteria concentration. It was found that higher inhalable dust concentrations produce a higher concentration of endotoxins (16, 19). The inhalable dust is mostly created during the aeration process in addition to the presence of inhalable dust, endotoxins, bioaerosols, and H_2_S. Other chemical substances might contribute to developing respiratory symptoms among sewage workers, such as NH_3_, NO, HCHO, and SO_2_. Nonetheless, only one study managed to prove their presence and its effect on sewage workers' respiratory health (25).

Moving to the environmental assessment studies done at sewage plants, the researchers wanted to measure the concentration of the potential hazards at sewage plants and not determine the effect of the exposure on the subjects' respiratory health. Most of these studies used stationary air samplers rather than personal air samplers to quantify potential respiratory hazards. H_2_S, bioaerosols (bacteria, fungi, and virus), PM 2.5, and VOC derivatives were mainly detected in the air at sewage plants. We found that more than half of environmental assessment studies in this systematic review need to consider the air samples in different seasonal variations. Seasonal factors play a significant role in humidity changes that could cause an alteration in potential hazard concentration, mostly airborne bacteria. Also, job locations or sites are among the main factors associated with respiratory hazard concentrations in the air. For example, an enclosed-type sewage plant may have a higher concentration of bacteria than an open-air type of sewage plant.

The possible reasons behind this finding may be that the higher airflow rate, leading to higher dilution effects of the bacteria, subsequently may lower the airborne bacteria concentration in the air. Next, sewage workers working within the mechanical treatment process had a higher probability of being exposed to airborne bacteria. These findings show that the types of jobs are one of the principal associated factors that need to be considered to assess sewage workers' exposure levels.

To date, there has only been a small number of studies measuring H_2_S, PM 2.5, and VOC concentration in sewage plant air. All these substances are proven in other studies to cause significant adverse respiratory health effects in industrial or occupational environments (15, 21–23, 25, 54–57). For example, in a study to estimate health risk from VOC removal from WWTP in China (38), the authors found a significant public health risk of VOC exposure to the people who live nearby the WWTP. The concentration of VOC emission is the highest at WWTP, which could harm the WWTP workers' respiratory health. In the future, there is a rising need to study VOC and particulate matter exposure among sewage workers and its effects on respiratory health. We can further discover new findings and valuable input to enhance knowledge gaps in this area.

## Limitations

We encounter several limitations. First we were only able to identify a small number of studies related to the relationship between potential occupational hazard exposure at sewage plants and respiratory health effects among sewage workers. Thus, it is difficult to find a higher number of high-quality studies pertaining to this field. Next, we were unable to conduct a statistical meta-analysis study due to the variety of methodologies that have been applied in the studies, and this could have produced a more reliable conclusion. Although we have done a quite extensive literature search from five online databases and used inclusive search terms, we were unable to rule out the likelihood of failing to spot some relevant articles.

## Conclusion

In conclusion, we found diverse literature in terms of study designs and results in this review. Overall, we need to accept an abundance of occupational respiratory hazard substances found at sewage plants, which could heighten the risk of developing adverse respiratory health effects among sewage workers. Several occupational respiratory hazards identified at sewage plants include H_2_S, bioaerosols (endotoxins, fungi, bacteria, and virus), PM 2.5, and VOC. Nonetheless, a few studies produce insignificant or mixed results. Hence, there is still a great need to conduct additional studies in the future to identify potential new exposures of occupational respiratory hazards. These studies would also require more vigilant methodologies to clarify the short- and long-term respiratory health effects.

## Data Availability Statement

The original contributions presented in the study are included in the article/supplementary material, further inquiries can be directed to the corresponding author/s.

## Author Contributions

KM conceptualized this study, reviewed the literature, assessed the literature quality, and drafted the article. SMY, ZI, and ARI proposed an article outline, reviewed the literature, assessed the literature quality, and revised and polished the article. All authors contributed to the article and approved the submitted version.

## Conflict of Interest

The authors declare that the research was conducted in the absence of any commercial or financial relationships that could be construed as a potential conflict of interest.

## Abbreviations

CNS: Central nervous system
COPD: Chronic obstructive pulmonary disease
DALY: Disability-adjusted life years
EPHPP: Effective Public Health Practice Project
ELISA: Enzyme-linked immunosorbent assay
FEV%: Forced expiratory volume percent of predicted value
FEV1: Forced expiratory volume in 1s
FVC: Forced vital capacity
HAdv: Human adenovirus
H_2_S: Hydrogen sulfide
HCHO: Formaldehyde
NH_3_: Ammonia
NO: Nitric oxide
NO_2_: Nitrogen dioxide
PCR: Polymerase chain reaction
PEF%: Peak expiratory flow percent of predicted value
PICO: Population, Intervention, Comparison and Outcomes
PM 2.5: Particulate matter 2.5
PPE: Personal protective equipment
PRISMA: Preferred Reporting Items for Systematic Reviews and Meta-Analyses
QMRA: Quantitative Microbial Risk Assessment
SO_2_: Sulfur dioxide
SR: Systematic review
STP: Sewage treatment plant
VOC: Volatile organic compound
WWTP: Wastewater treatment plant.
